# Ultrasonographic Insights into Peripheral Psoriatic Arthritis: Updates in Diagnosis and Monitoring

**DOI:** 10.3390/jpm14060550

**Published:** 2024-05-22

**Authors:** Karina Rossi Bonfiglioli, Fernanda Oliveira de Andrade Lopes, Letícia Queiroga de Figueiredo, Luis Fernando Fernandes Ferrari, Lissiane Guedes

**Affiliations:** Reumatology Division, Hospital das Clinicas da Faculdade de Medicina, Universidade de Sao Paulo, Sao Paulo 05403-010, Brazil; fernanda.oalopes@hc.fm.usp.br (F.O.d.A.L.); l.figueiredo@hc.fm.usp.br (L.Q.d.F.); luis.ferrari@hc.fm.usp.br (L.F.F.F.); lissiane.guedes@hc.fm.usp.br (L.G.)

**Keywords:** psoriatic arthritis, ultrasonography, synovitis, enthesitis, tenosynovitis, dactylitis

## Abstract

Psoriatic arthritis (PsA) is a chronic inflammatory arthritis associated with psoriasis, characterized by heterogeneous clinical manifestations and variable disease progression. Ultrasonography has emerged as a valuable tool in the diagnosis and monitoring of PsA, providing real-time visualization of joint and soft tissue abnormalities. This review highlights recent advancements in ultrasonographic techniques for the assessment of PsA, including the identification of typical features, the role of power Doppler imaging in detecting active inflammation, and the potential of ultrasound for guiding treatment decisions. Additionally, we discuss the utility of ultrasound in assessing treatment response and monitoring disease progression in patients with PsA, with a focus on novel imaging modalities. By elucidating the evolving role of ultrasonography in PsA management, this article aims to enhance clinicians’ understanding of its utility in facilitating early diagnosis, optimizing treatment strategies, and improving patient outcomes.

## 1. Introduction

Psoriatic arthritis (PsA) is a heterogeneous condition characterized by several distinct manifestations that can be present with varying degrees of severity simultaneously, posing challenges to both diagnosis and monitoring. Moreover, the different patterns of involvement of PsA can mimic other inflammatory arthritis, making thorough investigation crucial [1]. Peripheral joint disease includes polyarticular, oligoarticular, distal, and mutilans subtypes. Recognizing these patterns is necessary for the prompt diagnosis of PsA [2]. Once diagnosed, it is essential to conduct a comprehensive assessment of the disease, encompassing arthritis, enthesitis, dactylitis, skin and nail disease, and axial involvement [1]. Additionally, research indicates a pre-diagnostic phase in psoriasis (PsO) patients, marked by nonspecific musculoskeletal symptoms, preceding PsA onset [3], and there are no current biomarkers that allow for the early detection of the disease. In this context, ultrasonography has emerged as a sensitive tool for early PsA diagnosis and management, offering accessibility, cost-effectiveness, and real-time visualization of inflammatory changes. This review provides an overview of recent advancements in ultrasonographic techniques for the diagnosis and monitoring of peripheral PsA, focusing on key findings and clinical implications.

## 2. Ultrasonographic Assessment of Psoriatic Arthritis

### 2.1. Optimizing Machine Parameters and Techniques for Best Assessment

According to the 2017 EULAR standardized procedures for ultrasound imaging in rheumatology [4], US should be performed with high-resolution linear transducers. Frequencies should vary between 6 and 14 MHz for deep/intermediate areas and ≥15 MHz for superficial areas.

The main ultrasound modes are B-mode (or grayscale) and Doppler mode (color Doppler or power Doppler). B-mode provides morphological details of anatomical structures, while Doppler mode evaluates blood flow. Combining these modes is crucial for accurate assessment.

Several image optimization techniques are important to consider. In B-mode, the probe should be oriented perpendicular or parallel to the bony margin, as it appears bright (hyperechoic) and sharp. The area of interest should be explored by a dynamic scanning technique, to allow the best visualization of the structure. A common pitfall, particularly in tendons, is anisotropy (hypoechoic/anechoic appearance of a normally hyperechoic structure). To avoid this effect, the probe should be continuously adjusted to maintain the beam perpendicular to the tendon fibers, especially in insertional regions.

When using Doppler, it is important to maintain the lowest wall filter and an adequate frequency to the tissue’s depth. The gain should be adjusted by increasing it until random noise is seen in the image, then gradually lowering until only a few noise pixels are seen in the image. An adequate amount of gel must be seen in the top of the image, since excessive pressure can interfere with blood flow and Doppler detection.

In the assessment of peripheral psoriatic arthritis (PsA), particularly when examining small joints and entheses, special attention must be given to some of the above-mentioned aspects. Anisotropy can lead to misinterpretation of tendon and enthesis pathology, making it essential to consider this phenomenon to avoid diagnostic errors. Additionally, the evaluation of distal entheses of the fingers and nails benefits significantly from the use of high-frequency transducers, with those operating at frequencies of 18 MHz or higher providing the best detailing. Furthermore, Doppler adjustments should be optimized to enhance sensitivity for detecting low-velocity flows, which includes setting low-wall filters and using a low pulse repetition frequency. By carefully addressing these factors, examiners can improve the accuracy and effectiveness of their PsA assessments [5].

### 2.2. Synovitis

Among the articular changes observed in psoriatic arthritis (PsA), synovitis stands out as a hallmark feature, with prevalence rates ranging from 10% to 100% in previous ultrasound studies [6,7,8,9].

It has been reported also as an early finding in asymptomatic patients with psoriasis, suggesting a subclinical or pre-clinical stage of psoriatic arthritis [10,11].

The pathophysiology of PsA synovitis relies upon the concept of synovio-entheseal complex involvement [12]. It is hypothesized that mechanical stress on the enthesis, together with immunological predisposition, leads to enthesitis, represented histologically as bone marrow prominent edema, and adjacent immunological inflammation of the synovium.

Despite acknowledged clinical and pathogenic distinctions between PsA and RA [13,14], ultrasonographic evaluation and grading adhere to the definition established by the Outcome Measures in Rheumatology (OMERACT) ultrasound working group, initially developed for rheumatoid arthritis (RA). Synovitis is therefore defined according to OMERACT as the presence of synovial membrane hypertrophy (abnormal hypoechoic synovial tissue within the capsule, not displaceable and poor compressible) and may present a power Doppler signal. Grayscale synovitis is classified as grade 0 = no synovitis; grade 1 = minimal synovitis (below or at the level of the bony joint line); grade 2 = moderate synovitis (above the level of the bony joint line but without full distension of the joint capsule); grade 3 = severe synovitis (above the level of the bony joint line with distension of the joint capsule, which will appear convex). Power Doppler (PD) synovitis is classified as grade 0 = no flow within the synovium; grade 1 = up to three single spot signals, up to two confluent spot signals, or one confluent spot up to two single spot signals; grade 2 = PD signals covering <50% of the area of the synovium; grade 3 = PD signals in >50% of the area of the synovium.

Although there are also ultrasonographic similarities, including the same scores adopted for both, the primary distinctions arise in the pathophysiological realm, with the concept of synovio-entheseal complex involvement. In US, enthesopathy and peritendon inflammation (including tenosynovitis and paratendinitis) are more frequent in PsA joints with synovitis than in RA, as described by most studies [15,16]. Abdelghani KB et al. [17], however, could not demonstrate statistical differences between findings of peritendon inflammation in RA and PsA patients.

Besides the strong relation of tenosynovitis and enthesitis with PsA synovitis, other extra-articular findings are more frequent in these patients, including soft tissue edema, onychopathy, concurrent bone erosions, and neoformation within the joint [12,18], while in rheumatoid arthritis, inflammatory manifestations are primarily concentrated in areas rich in synovial tissue.

Regarding the amount of committed joints, PD positive synovitis in US is more frequent in RA, to the detriment of PsA [17,19], with no statistical difference for GS synovitis alone. In terms of joints affected, synovitis in RA often is present in multiple joints symmetrically, with metacarpophalangeal involvement being more frequent than in PsA. In PsA, conversely, it predominantly involves the proximal and distal interphalangeal (PIP and DIP) joints [17,18,20].

### 2.3. Enthesitis

The enthesis is the site of attachment of tendons, ligaments, and joint capsules to the bone [21]. It serves as a crucial connection point between the soft, force-generating tissues (such as muscles) and the solid framework of the body, such as the bones. They can be classified histologically as fibrous (located at the diaphysis or metaphysis of long bones) or fibrocartilaginous (located at the epiphyses or apophyses). Clinically, fibrocartilaginous entheses represent the characteristic target in patients with seronegative spondyloarthritis (SpA), including psoriatic arthritis (PsA) [22].

Enthesitis is currently regarded as a fundamental pathological process in the development of SpA and a key finding of musculoskeletal inflammation in diseases from the SpA spectrum, such as PsA. This condition leads to significant inflammation, bone destruction, and pathological bone proliferation, highlighting its critical role in the progression and severity of these diseases [23].

Diagnosing enthesitis presents significant complexities, particularly in the early stages. Despite its prevalence, clinical examination only detects it in around 30% of patients with PsA [24]. This low detection rate stems from various factors, including the deep-seated nature of entheses, the subtle manifestation of symptoms, and the potential overlap with conditions like central sensitization and fibromyalgia. Classic signs of inflammation are often absent. Given the challenges in identifying enthesitis through routine clinical assessment [25], there has been a growing interest in using imaging modalities in this context. Conventional radiography (CR) is readily available and useful for detecting bone damage but has limited value in identifying enthesitis. In contrast, magnetic resonance imaging (MRI) offers a thorough evaluation of enthesitis. Nevertheless, its utilization is hindered by significant drawbacks, notably its high cost, especially in resource-limited settings, and the prolonged time required to examine multiple targets.

In this context, ultrasonography (US) has the potential to become the gold standard for the entheseal assessment, particularly in the early stages. US offers real-time imaging with a comprehensive view of the synovial–enthesis complex, revealing morpho-structural and vascular abnormalities that indicate both active inflammation and structural damage at the entheseal site [26]. Both B-mode and Doppler US offer valuable assistance in identifying subclinical enthesitis and precisely quantifying disease activity [27,28,29].

**Figure 1:**
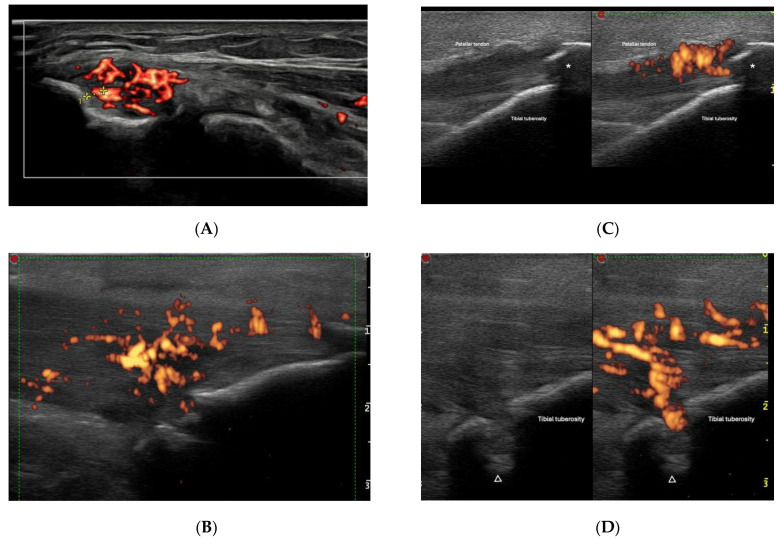
(**A**) shows the lateral epicondyle longitudinal aspect, with the power Doppler signal in the common extensor tendon (within 2 mm of the cortical bone). The longitudinal aspect of patellar tendon in its tibial insertion is shown in (**B**–**D**). Tendon thickening, hypoechoic appearance, and the presence of power Doppler can be observed. Damage aspects, such as enthesophytes (marked with *) and bone erosion (marked with Δ) can be seen [(**D**) shows the same enthesis twice, in grayscale (left) and with power Doppler (right)].

**Figure 2:**
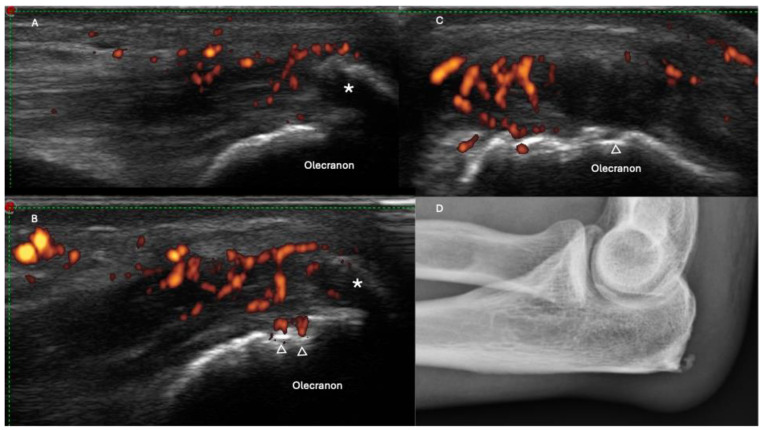
Triceps tendon longitudinal aspect (**A**–**C**) showing inflammatory changes, such as thickening, hypoechoic appearance, and the presence of power Doppler within 2 mm of the cortical bone. Damage findings such as enthesophytes (marked with *) and bone erosion (marked with Δ) can also be seen, in correlation with enthesophyte findings in an elbow X-ray (**D**).

Over the past two decades, the OMERACT US Task Force has dedicated significant efforts to improving the standardization of ultrasonography (US) assessments for entheseal pathologies [30,31,32]. A final US definition of enthesitis was determined as “hypoechoic and/or thickened insertion of the tendon close to the bone (within 2 mm from the bony cortex), which exhibits Doppler signal if active and which may show erosions and enthesophytes/calcifications as a sign of structural damage” (Table 1) (Figure 1 and Figure 2) [26,33].

With the increasingly advanced technology of ultrasound machines, capable of producing high-resolution images with high-frequency transducers, greater attention has been directed towards the “mini entheses” of the hands. Those include the insertion of the extensor apparatus of the fingers at the middle and distal phalanges, the insertion of the flexor apparatus of the fingers on the volar surface of the distal phalanges, and the insertions of the collateral ligaments of the fingers [16,17]. Inflammatory involvement of these insertions supports the diagnosis of PsA and enhances our understanding of anatomical contiguity processes. For instance, nail involvement can be related to enthesitis of the distal apparatus, illustrating the interconnected nature of these inflammatory processes.

### 2.4. Tenosynovitis, Peritendinitis, and Functional Enthesitis

Distinguishing between tenosynovitis and peritendinitis is critical; based on their anatomical characteristics, while both variants share similarities in their clinical presentation, their locations differ significantly. Tenosynovitis refers to inflammation of the tendon sheath, whereas peritendinitis occurs in tendons lacking a sheath, affecting the paratenon and the muscle–tendon junction [34]. They are both displayed in PsA.

Tenosynovitis is defined by OMERACT as abnormal anechoic and/or hypo-echoic (relative to tendon fibers) tendon sheath widening, which can be related both to the presence of tenosynovial abnormal fluid and/or hypertrophy. Power Doppler may or may not be present. A PD is defined by OMERACT as abnormal peritendinous Doppler signal within the widened synovial sheath, seen in two perpendicular planes, and also abnormal intra-tendinous signal in two perpendicular planes [35]. In clinical and ultrasonographic studies on PsA, tenosynovitis is the second most prevalent manifestation (after synovitis) and possibly the most significant contributor to symptoms. This is particularly evident in the involvement of the flexor tendons of the hands, a phenomenon observed even in patients solely diagnosed with PsO (5.3%) [36,37]. It is possible that the close anatomical relationship with annular pulleys, which works as a functional enthesis, may contribute to the development of flexor tenosynovitis [37].

The term “functional enthesis” was coined by Benjamin and McGonagle [22] to describe sites where tendons and ligaments encircle bony pulleys. In these regions, where hard and soft tissues converge without direct anchorage as seen in typical entheses, a significant interplay occurs. The presence of compressive forces at both the fibrocartilaginous enthesis insertion and the enveloping tendons results in a similar functional demand of the fibrocartilage. This observation may elucidate why this area is also susceptible to involvement in PsA, resembling the concept of the “Deep Koebner” phenomenon associated with accessory pulleys of the flexor tendons [38]. These pulleys may exhibit thickening and PD signal, likely attributable to repetitive microtrauma and at least partially comprising fibrocartilaginous tissue [38,39,40].

Less frequent yet highly characteristic, peritendonitis (PTI) serves as a significant differentiator from RA and is particularly specific to PsA [7]. This condition usually involves the hand’s extensor digitorum tendons and was originally described by Gutierrez et al. in 2010 [15], defined as “hypoechoic swelling of the soft tissue surrounding the extensor digitorum tendon, with or without peri-tendinous PD signal”. More recently, it has also been interpreted as a functional enthesitis, based on prior anatomical studies revealing the existence of fibrocartilage within the extensor tendon at the metacarpophalangeal joint level [40]. While PTI remains more specific to PsA and serves as a valuable marker aiding in the differentiation from early RA [16], it is also important to note that it has recently been described in systemic lupus erythematosus (SLE), palindromic rheumatism, and RA [41,42,43].

In summary, recent evidence regarding ultrasonographic changes involving hand tendons in psoriatic arthritis highlights the pivotal role of hand “mini-enthesitis” affecting surrounding tissues and contributing to a complex and heterogeneous pattern of inflammatory involvement.

### 2.5. Dactylitis

**Figure 3:**
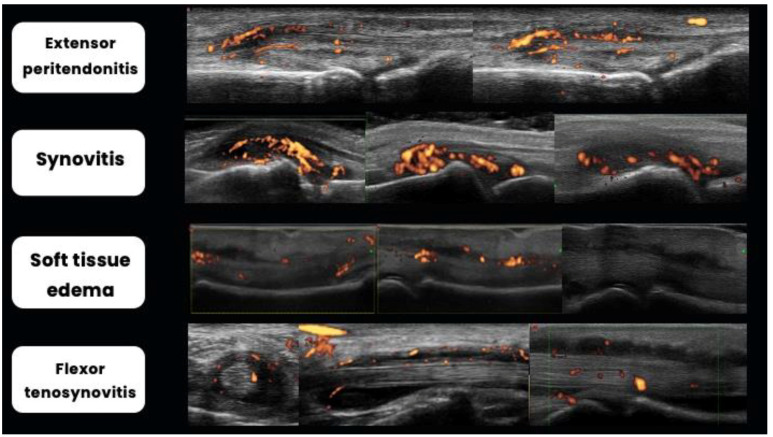
Pathological elements of dactylitis, according to Zabotti A et al. [48] and OMERACT [45] definitions. Extensor peritendonitis is shown longitudinally in the dorsal aspect of the proximal interphalangeal joint. Synovitis is shown longitudinally in the dorsal aspect of the metacarpophalangeal joint. Soft tissue edema is shown longitudinally in the palmar aspect of the proximal interphalangeal joint topography. Flexor tenosynovitis is shown transversally (**left**) and longitudinally (**right**) in the palmar aspect of the metacarpophalangeal joint topography.

Dactylitis, which is characterized by the uniform swelling of a finger, is a pivotal pathology of PsA, affecting nearly half of patients [44]. Often serving as the initial sign of disease onset, dactylitis can also present as the sole manifestation. Given that dactylitis entails the involvement of various anatomical structures [45,46,47], it can be challenging to discern each component solely through clinical evaluation, highlighting the significant role ultrasound plays in delineating their respective contributions. Moreover, US assessment promotes an additional benefit of examining inflammatory status using Doppler techniques. The Outcome Measures in Rheumatology (OMERACT) ultrasound group has proposed potential ultrasound inflammatory lesions indicative of dactylitis, including soft tissue thickening and edema, flexor tendon tenosynovitis, and joint synovitis [45]. In 2020, Zabotti A et al. [48] proposed a global sonography score for dactylitis in PsA, including composite scoring of peritendon extensor inflammation, soft tissue edema, flexor tenosynovitis, and EULAR-OMERACT combined score for synovitis (Figure 3). Validation and standardization of US assessment of dactylitis are still ongoing processes.

Ultrasound studies have revealed the frequency of each pathological component for clinical dactylitis. The most common lesions were soft tissue thickening (81%) and subcutaneous edema (74%), followed by synovitis (56–68%) and flexor tenosynovitis (52%). Doppler activity was most frequently detected subcutaneously (55%) and around the flexor tendons (45%). Typically, these lesions were found in combinations, with the most prevalent combination being subcutaneous edema and synovitis (71%) [49].

### 2.6. Bone Changes

**Figure 4:**
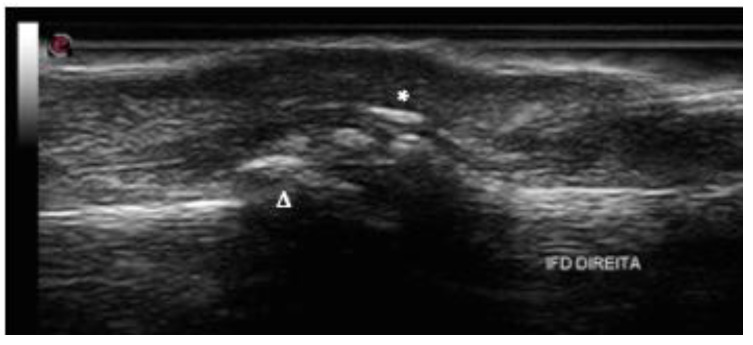
New bone formation (Δ) and enthesophytes (*) in distal interphalangeal joint dorsal longitudinal aspect.

Besides soft tissue findings, bone changes are significant findings in PsA, including erosions, enthesophyte, periosteal reaction, and new bone formation [50] (Figure 4). A notable characteristic that sets PsA apart from other arthritic conditions is the concomitant occurrence of bone proliferation and bone erosion in the same joint [8].

Recent data on osteoimmunology highlight that bone destruction and bone neoformation are direct consequences of enthesitis, a condition recognized as one of the fundamental pathological processes in the development of SpA. This understanding underscores the critical role of enthesitis in the progression of SpA and its impact on bone integrity and remodeling [23].

In SpA, the intricate mechanism of bone neoformation and erosion is characteristic of the disease’s pathology. Key regulatory factors in osteogenesis, including Bone Morphogenetic Proteins (BMPs), the Wnt signaling pathway, and Hedgehog (Hh) signaling, play crucial roles in this process. Furthermore, major cytokines such as IL-17, TNFα, IL-23, and IL-6 are deeply involved in the pathogenesis and amplification of inflammation within the SpA disease spectrum. These cytokines interact with the bone microenvironment, contributing to both the erosive and proliferative bone changes that characterize the disease [23].

Regarding ultrasound, the Outcome Measures in Rheumatology (OMERACT) defines bone erosion as an intra-articular disruption of the bone surface visible in two perpendicular planes. Previous studies have reported varying prevalence rates of bone erosion in PsA, ranging from 10.8% to 52% [7,9,51,52]. It is classically described in joints along with adjacent bone proliferation. However, with the increasing use of US to evaluate enthesitis and articular changes in PsA, erosions in enthesis are also frequent findings in this group of patients. They are described as indicative of aggressive behavior of PsA at the joint level, with a marked effect on subsequent structural damage, mainly if associated with PD on enthesis insertion [53,54].

**Figure 5:**
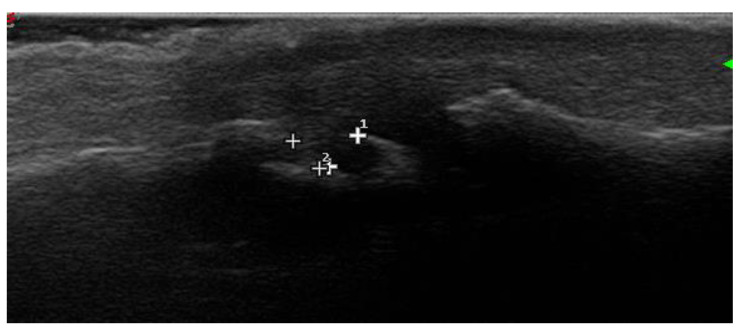
Erosions in proximal interphalangeal joint dorsal longitudinal aspect.

Despite the OMERACT definition used being the same for other inflammatory arthropathies, specific ultrasonographic findings may help differentiate PsA from the others. Unlike rheumatoid arthritis (RA), where bone erosions predominantly affect the metacarpophalangeal (MCP) and proximal interphalangeal (PIP) joints, in PsA, they are reported as being more common in the distal interphalangeal (DIP) and PIP joints [8] (Figure 5) [55,56], whereas Smerilli G et al. [50] described them as being more frequent in the fifth metatarsophalangeal joint when compared to MCF and the ulnar head. Additionally, research suggests that bone erosions in PsA tend to be smaller and less frequent compared to RA [19,55].

### 2.7. Nail Changes

Nail psoriasis occurs in 15% to 50% of patients with psoriasis and 41% to 93% of patients with PsA [57,58]. Anatomically, the nail consists of four main components: the matrix, the nail bed, the hyponychium, and the eponychium. These structures are closely positioned to the distal phalanx, anchored by Sharpey fibers that insert into the bone in a manner similar to an enthesis. In addition, the extensor tendon enthesis inserts into the area adjacent to the nail root [59,60,61]. The association between nails and joints is particularly marked in the presence of DIP joint arthritis, and 80% to 100% of such patients have nail involvement that frequently occurs at the adjacent nail. Its involvement has been reported to be a predictor of development of PsA in patients diagnosed with PsO [62,63]. Ultrasound changes can provide predictive insights prior to the visible onset of macroscopic changes (such as leukonychia, nail pitting, crumbling, red spots, onycholysis, salmon or oil-drop patches, subungual hyperkeratosis, and splinter hemorrhages), described in the Nail Psoriasis Severity Index (NAPSI).

Nail unit structures have different densities that enable their differentiation through ultrasound. The nail is formed by the nail unit and the periungual zone. The first one is composed of nail plates, the nail matrix, and the nail bed, and the periungual zone is composed of the periungual tissues or periungual folds [64]. The nail plate is a trilaminar structure that originates at the middle third of the phalanx. It is composed of two parallel hyperechoic bands, named ventral and dorsal plates (with thickness ranging between 0.3 and 0.65 mm), separated by a virtual space denominated the interplate space that has a hypoechoic/anechoic appearance [65].

The nail matrix is a hyperechoic structure, located in the proximal aspect of the nail bed, and its length varies between 1 and 5.3 mm, being denominated NMT [65]. The nail bed is a hypoechoic structure located immediately inferior to the nail plates extending to the periosteum of the distal phalanx. The average thickness is 1.5 mm (0.7–6 mm), which must be measured in the middle third of the phalanx and represented by NBT [65]. The periungual tissues are divided depending on their location as proximal fold (eponychium), lateral folds (perionychium), or distal fold (hyponychium). The phalanx is the deepest structure, appearing as a hyperechoic band, located immediately inferior to the nail bed.

**Figure 6:**
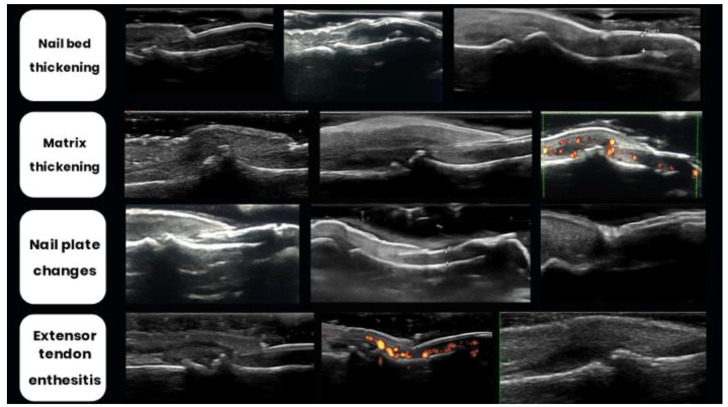
Typical morphological nail and enthesis changes in psoriatic arthritis, as described by Worstman [62,64], Cunha [65], and Naredo [66].

Ultrasound (US) changes (Figure 6) are primarily characterized by the morphological descriptions outlined by Worstman et al. [64,66]. Revisiting the concept of enthesitis as the initial milestone and recognizing the nail’s connection to this structure, psoriatic arthritis (PsA) manifests with initial alterations beginning in nail bed thickening (NBT, typically > 2.5 mm). Subsequently, there is a loss of definition of the ventral plate followed by the fusion of both ventral and dorsal plates, resulting in the disappearance of the intermediate anechoic layer [67].

Differing from the majority of anatomical sites, under normal conditions, a minimal quantity of blood flow can be detected within the nail bed (due to the presence of thin digital arterial and venous vessels). When an onychopathy is present, this blood flow increases (easily detectable by PD) [15]. Naredo et al. [68], inspired by Arbault et al. [69], proposed the power Doppler (PD) classification of the nail bed and nail matrix with a score between 0 and 3, according to the PD enhance distribution pattern. (grade 0: no PD signal; grade 1: confluent PD signal in <25% of the area; grade 2: PD signal in 25 to 50% of the area; grade 3: PD signal in >50% of the area)

Combining both classifications, Cunha et al. [67] proposed the index Brown University Nail Enthesis Scale (BUNES), which evaluates the morphology of three areas: the nail matrix (A), the nail plate (B), and the nail bed (C), scoring 0 (normal) or 1 (abnormal or thickened), and the presence of PD (Table 2).

Moreover, as already reviewed, the OMERACT definition of enthesitis has been applied to the periungual tissues, particularly to the extensor tendon of the digits originating from the distal phalanx.

## 3. Role of Ultrasonography in Diagnosis, Monitoring, and Treatment Assessment

The diagnosis of psoriatic arthritis (PsA) primarily relies on clinical features, yet in uncertain cases or during early disease stages, the integration of joint and periarticular ultrasound is increasingly recognized as a valuable diagnostic adjunct. It has been demonstrated that ultrasound (US) findings exhibit high concordance with magnetic resonance imaging (MRI) results, offering the advantage of lower cost and easier accessibility compared to MRI [70].

Firstly, ultrasound aids in distinguishing PsA from other inflammatory arthropathies through distinctive ultrasonographic patterns. Notably, studies have highlighted the higher specificity of extra-articular manifestations such as enthesitis, soft tissue edema, peritendinitis, dactylitis, and nail changes in PsA, contrasting with the predominantly articular findings observed in conditions like rheumatoid arthritis (RA) [17,18,56].

Expanding beyond diagnostic utility, ultrasound (US) assessment also holds prognostic significance. Ultrasound assists in identifying early inflammatory markers in psoriasis patients at elevated risk of developing PsA over time [71]. It is hypothesized that psoriasis patients pass through an asymptomatic subclinical phase with articular and periarticular inflammation [72], followed by a symptomatic phase not fulfilling the CASPAR diagnosis criteria, and at last established PsA with symptoms and articular and periarticular inflammation.

Corroborating this concept, cross-sectional studies suggest that synovitis, tenosynovitis, erosions, and enthesitis on ultrasound are more frequent in asymptomatic psoriasis patients than controls [11,73,74]. Regarding the risk of prospectively evolving from asymptomatic into established PsA, data diverge in the literature. The presence of enthesitis (mainly with enhanced vascularity) and higher prevalence and grade of synovitis (on grayscale and power Doppler) are the most frequently described US findings associated with progression to PsA [75,76]. Other findings such as onychopathy at baseline as well as persistent synovial inflammation are described as risk factors as well [76].

Besides predicting progression from psoriasis to PsA, US findings can also foresee radiographic progression of arthritis. Research shows that US-detected enthesopathy in PsA correlates with increased radiographic damage, both in axial and peripheral joints, indicating its value in predicting disease progression [77,78]. Polachek et al. [79] found a positive relationship between higher scores on the Madrid Sonographic Enthesitis Index (MASEI) and increased radiographic joint damage in the hands and feet, as evaluated by the modified Steinbrocker score (mSS), as well as correlation of elevated MASEI values with severe manifestations like arthritis mutilans. Similarly, dactylitis has been associated with erosive disease [80].

Therefore, ultrasonographic assessment in psoriasis patients has been a subject of increasing interest. The rationale lies in the potential for more periodic clinical reassessment in this group of patients known to have a higher risk of psoriatic arthritis (PsA). This proactive approach could facilitate an earlier diagnosis, ultimately preventing damage.

## 4. Conclusions

Ultrasonography has revolutionized the management of PsA by providing real-time visualization of inflammatory changes in affected joints and tissues. Recent advancements in ultrasonographic techniques have enhanced our understanding of the pathophysiology of PsA and facilitated early diagnosis, accurate assessment of disease activity, and monitoring of treatment response.

Some characteristic ultrasound findings may be present in the diagnosis of psoriatic arthritis (PsA), including synovitis, peritendinitis, tenosynovitis, enthesitis, “mini enthesitis” of the hands, dactylitis, periarticular/peritendinous soft tissue edema, and nail disease. By incorporating ultrasound into routine clinical practice, clinicians can optimize treatment strategies, improve patient outcomes, and mitigate the long-term burden of PsA. Future research efforts should focus on standardizing ultrasound protocols, validating quantitative measures, and exploring the role of novel imaging modalities.

## Figures and Tables

**Table 1:** Inflammatory and damage domains from the OMERACT (Outcome Measures in Rheumatology) enthesitis definition [23].

Inflammatory Domain	Damage Domain
Power Doppler sign (within 2 mm of the bone)	Erosions
Hypoechogenicity	Enthesophytes/calcifications
Thickening	

**Table 2:** BUNES (Brown University Nail Enthesis Scale) classification of the psoriatic nail, according to morphological and power Doppler domains.

BUNES Classification		
Variables	Morphometry	Power Doppler
A (nail matrix)	0/1	0/1/2/3
B (nail plate)	0/1	0/1/2/3
C (nail bed)	0/1	0/1/2/3
Max score	3	6

## Data Availability

Not applicable.
